# Ion mobility conformational lipid atlas for high confidence lipidomics

**DOI:** 10.1038/s41467-019-08897-5

**Published:** 2019-02-28

**Authors:** Katrina L. Leaptrot, Jody C. May, James N. Dodds, John A. McLean

**Affiliations:** Center for Innovative Technology, Department of Chemistry, Vanderbilt Institute of Chemical Biology, Vanderbilt Institute for Integrative Biosystems Research and Education, Vanderbilt-Ingram Cancer Center, Vanderbilt University, Nashville, TN 37235 USA

## Abstract

Lipids are highly structurally diverse molecules involved in a wide variety of biological processes. Here, we use high precision ion mobility-mass spectrometry to compile a structural database of 456 mass-resolved collision cross sections (CCS) of sphingolipid and glycerophospholipid species. Our CCS database comprises sphingomyelin, cerebroside, ceramide, phosphatidylethanolamine, phosphatidylcholine, phosphatidylserine, and phosphatidic acid classes. Primary differences observed are between lipid categories, with sphingolipids exhibiting 2–6% larger CCSs than glycerophospholipids of similar mass, likely a result of the sphingosine backbone’s restriction of the sn1 tail length, limiting gas-phase packing efficiency. Acyl tail length and degree of unsaturation are found to be the primary structural descriptors determining CCS magnitude, with degree of unsaturation being four times as influential per mass unit. The empirical CCS values and previously unmapped quantitative structural trends detailed in this work are expected to facilitate prediction of CCS in broadscale lipidomics research.

## Abstract (web summary)

The biological functions of lipids critically depend on their highly diverse molecular structures. Here, the authors determine the mass-resolved collision cross sections of 456 sphingolipid and glycerophospholipid species, providing a reference for future structural lipidomics studies.

## Introduction

Lipids are an essential class of biomolecules, performing functions such as contributing to cell membrane structure, regulating cell activities, and storing concentrated energy, among others^1–3^. Lipids represent a wide array of structurally diverse, often isomeric, molecules due to the fact that each lipid can vary in headgroup type, acyl chain length, position of attachment, degree of unsaturation, and stereochemistry^4^. The position of double bonds in lipids is important in the determination of their biological function; for example, naturally occurring conjugated linoleic acid (CLA) isomers have been revealed to play varied biological roles based on the positions of the double bonds in the acyl tail. Specifically, the effects of *trans*-10,*cis*-12 CLA on body composition and *cis*-9,*trans*-11 CLA on growth/feed efficiency appear to be a result of separate biochemical mechanisms^5^, and only the *trans*-10,*cis*-12 CLA isomer regulates human stearoyl-CoA desaturase in HepG2 cells^6^.

Lipid research from the 1960s and 1970s contributed much of our current knowledge of lipid biochemistry and metabolism^7^ despite the fact that, in the past few decades, several new analytical techniques have emerged to elucidate lipid structural details, specifically in the field of mass spectrometry (MS). For example, structures of brain gangliosides have been investigated by tandem MS/MS utilizing low-energy collision induced dissociation (CID)^8^ as well as by high resolution Fourier-transform MS with chip-based nano-electrospray ionization^9^. Post-source decay fragment ion analysis has been utilized to assign the position and identity of fatty acid residues on the glycerol backbones of glycerophospholipids^10^, and recently an implementation of electron induced dissociation, known as electron-impact excitation of ions from organics (EIEIO), has been used to produce extensive untargeted acyl chain fragmentation and allows for determination of both sn-position and double bond location in lipid standards^11^. Additional techniques that promote carbon double bond cleavage have been developed in conjunction with MS to identify double bond position, in particular the Paternò-Büchi reaction^12^ and ozonolysis^4,13–16^.

An emerging analytical technique that has found utility in lipid structural analysis is ion mobility coupled to MS (IM-MS)^11,17–25^, which is selective to gas-phase structure. IM measures the gas-phase mobility of analytes which can be used to calculate a 2-dimensional collision cross section (CCS), an averaged measurement of the cross sectional area of the analyte ion in the gas phase. Thus, IM separates lipid molecules based on differences in their CCS, and interfacing IM with MS results in a comprehensive 2D separation capable of differentiating isomers and delineating molecules into respective biomolecular classes^26,27^.

Detailed IM-MS analyses have previously revealed specific and reproducible mobility-mass correlations within each biomolecular class, related to molecular structures and packing efficiencies^28–30^. Precise and reproducible lipid CCS measurements can allow for quantitative descriptions of these previously observed structural trends to be developed, which in turn can enable predictive models to be used in support of lipid identification and characterization^28,31^. Newly developed IM-MS instrumentation based on high-precision, uniform field measurements has enabled the quantitation of trends which have been previously observed with other IM-MS methods^31,32^, in smaller data sets^17,18,23,33,34^, and in broader training data sets to predict CCS values^32,35,36^.

In this study, we focus on the relationship between lipid structure and gas-phase conformation via IM-MS analysis. These trends manifest in each lipid class and relate to conformational changes as a result of increasing the degree of unsaturation or acyl chain lengths. This work quantifies correlations directly between mass and empirical CCS values important for lipid feature identification in lipidomics analyses.

## Results

### Lipid nomenclature

Lipid nomenclature in this study follows the classification system used in LIPIDMAPS (http://www.lipidmaps.org) and developed by Fahy, et al.^37,38^, where the first set of letters represents the lipid class (head group), modifications are denoted by any following letters (h or HETE for hydroxyl group presence and O for loss of a carboxyl group from one of the fatty acyl chains), the number preceding the colon denotes the summed carbon chain lengths, and the number following the colon refers to the total number of double bonds in the carbon chains. Only singly-charged, monomeric ions are reported here, and there is no evidence in the spectra of lipid monomers adopting higher charge states. Although multiply charged multimers are also observed in low abundance, the majority of these are heteromultimers arising from combinations of different lipids, which are difficult to identify using single-stage IM-MS alone.

Lipids were analyzed from class-specific TLC fractions and identifications were assigned primarily based on exact mass measurements. The CCS measurements and corresponding mobility-mass correlations were utilized to provide additional confidence in the lipid assignment in conjunction with the mass measurement accuracy (±10 ppm observed in this work).

### Lipid population observations

**Fig. 1:**
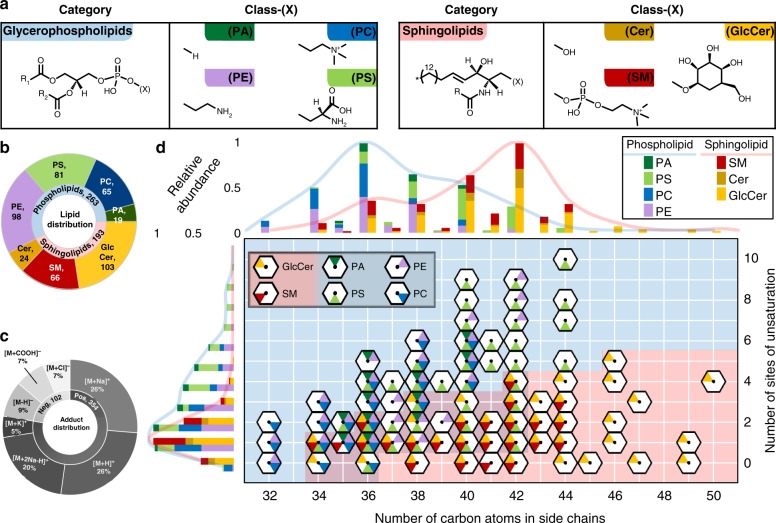
Lipid population observations. **a** Investigated lipids are classified by head group. Expected and observed species are listed by the total number of carbons or double bonds in the fatty acid tails. **b** The distribution of observed lipids. The inner ring denotes lipid categories whereas the outer ring details features observed in each lipid class. **c** The distribution of adducts observed resulting in the species of quasi-molecular ion detected. **d** Central graph represents population of lipid summed chain lengths (*x*-axis) and degrees of unsaturation (*y*-axis) identified, separated by class and excluding adduct and modification information. The Cer class of lipids are included with GlcCer. Each colored wedge of the hexagons represents an identified lipid as indicated in the key. Background shading delineates lipid categories, with sphingolipids in red and glycerophospholipids in blue. Left histogram displays distribution of degree of unsaturation, normalized to each category. Top histogram shows summed chain length distribution, normalized to each category

This work presents 456 CCS values for 217 uniquely identified lipids representing 7 lipid classes (Fig. 1a, Supplementary Data 1) analyzed by uniform field IM-MS in both positive and negative ionization mode. These measurements were obtained on a drift tube instrument operated with nitrogen drift gas (^DT^CCS_N2_). Lipid categories included glycerophospholipid (PA, PE, PC, and PS) and sphingolipid (Cer, GlcCer, and SM) extracts from chicken egg and porcine brain. The resulting lipid identification distributions are presented in Fig. 1b. For glycerophospholipids, it was found that longer alkyl chain lengths allowed accommodation of more sites of unsaturation, with PA and PC species containing as many as 6 sites of unsaturation, and PE and PS including as many as 9 and 10 sites of unsaturation, respectively. Observed sphingolipids had less sites of unsaturation, as is common in biological samples, with 5, 4, and 3 or less doubly bonded carbons for GlcCer, SM, and Cer, respectively. Maximum alkyl chain lengths increased with the head group size for glycerophospholipids, with PA lipids found with 35–40 carbons, PE 32–42, PC 32–40, and PS 34–44. The sphingolipids exhibited a slightly wider range of chain lengths than the glycerophospholipids, with GlcCer having 34–50 carbon atoms, SM 34–44, and Cer 36–44. These observations are summarized in the histograms of Fig. 1d.

### Category and class IM-MS correlation

**Fig. 2:**
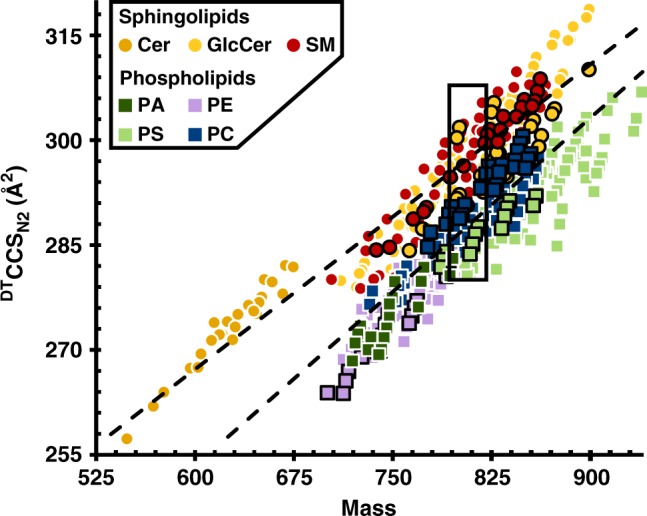
Conformational space analysis of all 456 singly charged lipids surveyed in this work. Included are data from 7 classes from 2 lipid categories in positive and negative ionization mode. Markers for cations are indicated by a white border while those for anions are indicated by a black border. Error bars represent standard errors and are for each point within the scale of the marker. Specific lipid identifications, numerical measurement data, and number of measurements per data point (*n* = 8 or 16 repeat measurements for positive ionization mode data, *n* = 20, 21, or 28 repeat measurements for negative ionization mode data) are provided in Supplementary Data 1

Previous IM-MS studies have demonstrated that biomolecules separate into distinctive class-based correlations in plots of CCS vs. mass^27^, and that different lipid categories, e.g., glycerophospholipids and sphingolipids, occupy unique space within these correlations^28,31^. In this work, all primary lipid classes exhibit a positive mobility-mass correlation in conformational space analyses. Within the combined lipid trendline, unique lipid categories (sphingolipids and glycerophospholipids) could be further differentiated, with little overlap, by their respective ^DT^CCS_N2_ information, as each category exhibited an average CCS increase of 0.15–0.17 Å^2^ per mass unit, with glycerophospholipids having ^DT^CCS_N2_ values 6–16 Å^2^ (2–6% difference) less than sphingolipids of equivalent mass in the investigated mass range of 500–1000 Da (Fig. 2, Supplementary Table 1). This finding suggests that lipids originating from complex mixtures can be readily classified into one of these two primary lipid categories using CCS and mass information.

**Fig. 3:**
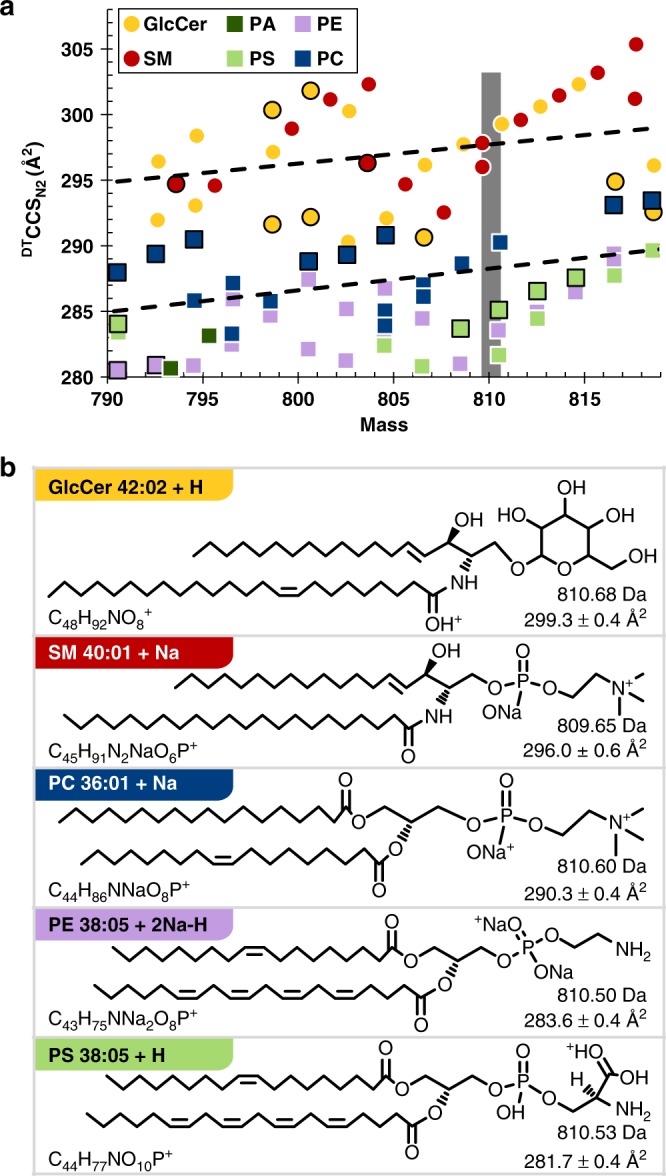
Conformational space occupancy of lipids within a narrow range of mass. **a** Expanded region from Fig. 2 highlighting occupancy of each lipid class by mass and ^DT^CCS_N2_. Note the largest ^DT^CCS_N2_ difference for peaks of similar mass is between glycerophospholipids and sphingolipids. Markers for cations are indicated by a white border while those for anions are indicated by a black border. Error bars represent standard errors and are within the marker itself for each point. **b** Primary structures of the five positively charged nominal mass isomeric lipid ions (809.6–810.7 Da, SM structure is for the feature of lesser cross section) highlighted in panel A. ^DT^CCS_N2_ values are all statistically different and largest for sphingolipids. Note structural information is inferred from rules described by Voet, et al. with (i) lipids assembled as concatenations of C_2_ units making even-numbered chains prevalent, (ii) the first unsaturation site preferably located between C9 and C10, (iii) subsequent unsaturation sites occurring every third bond, and (iv) double bonds existing primarily in the cis- configuration^44^. Poly-unsaturated fatty acyl side chains are constructed from commonly encountered fatty acid structures with preference for ω-3 and ω-6 chains. Adducts are shown at likely basic sites. Additionally, sphingolipids contain a sphingosine backbone. Specific lipid identifications, numerical measurement data, and number of measurements per data point (*n* = 8 or 16 repeat measurements for positive ionization mode data, *n* = 20, 21, or 28 repeat measurements for negative ionization mode data) are provided in Supplementary Data 1

A closer examination of the IM-MS data (Fig. 3a) shows a regular increase in size (linear slopes) for individual lipid classes within the glycerophospholipid category with corresponding slopes (^DT^CCS_N2_ vs. mass) ranging from 0.15 to 0.18 Å^2^ per mass unit. Classes within the sphingolipid category exhibit a slightly larger increase in size with mass, with empirically observed slopes of 0.19 Å^2^ per mass unit. The larger conformations observed for sphingolipids are related to the limited degrees of unsaturation present in the sphingosine backbone. Lipid class trends within each category are very similar, indicating that while the acyl chain governs the change in CCS, the lipid head group dominates the overall magnitude of the ion cross section. This observation is discussed in detail below.

Although sphingomyelin is categorized as a sphingolipid due to the sphingosine backbone, as a ceramide with phosphocholine in the head group, it shares structural aspects with the PC lipid class, whereas other lipid classes investigated in this work fall strictly into a single lipid category by conventional definitions. Interestingly, although sphingomyelin contains structural attributes of both the sphingolipid and glycerophospholipid categories, instead of falling between PC and GlcCer, it exhibits larger than expected CCS values, being similar in size to GlcCer (Fig. 3a). This is likely due to a combination of the sphingosine backbone constraining the sn1 tail length and the choline head group conformation, which packs less efficiently than the GlcCer monosaccharide headgroup.

### Ion forms

In both positive and negative ionization modes, multiple adducts are commonly formed for each lipid species. In positive ionization mode, coordination of protons and sodium ions were predominantly observed in this study (Fig. 1c). Positive identifications with these common adducts prompted subsequent searches for the same lipids with other adducts to deconvolute the spectra. For example, SM 36:01 was subsequently found in the spectra as [M + H]^+^, [M + Na-H_2_O]^+^, [M + Na]^+^, and [M + K]^+^, while both PC 34:01 and PE 34:01 were found as [M + H]^+^, [M + Na]^+^, [M + K]^+^, and [M + 2Na–H]^+^. In negative ionization mode, loss of a proton or coordination of either chloride (Cl^-^) or formate (COOH^−^) anions were observed. While PE and PS lipids were only observed to ionize by the loss of a proton, PC and SM were only observed as [M + Cl]^−^ or [M + COOH]^−^, and GlcCer was observed as [M−H]^−^, [M + Cl]^−^, or [M + COOH]^−^.

Commonly observed cation coordination varied slightly between the different lipid classes with [M + Na]^+^ being most common, followed by [M + H]^+^ which was observed in all classes except PA. [M + 2Na–H]^+^ adducts were identified in SM and all four glycerophospholipid classes, and [M + K]^+^ features were identified in SM, PC, PE, and PS. The lack of appearance of the [M + K]^+^ adduct in PA is likely related to the lower abundance of this class. Neutral water loss, though common in the hydroxyl abundant sphingolipids, was not observed in glycerophospholipids: [M + H-H_2_O]^+^ occurred in Cer, GlcCer, and SM, and [M + H-2H_2_O]^+^ was observed in Cer and GlcCer. Whether water loss occurs in solution or during ionization is unknown.

The nature of the charge carrier was found to influence the overall CCS of all lipids, and the relative change in CCS across the different charge carriers is summarized in Supplementary Fig. 1. In general for positively charged ions, [M + Na]^+^, [M + K]^+^, and [M + 2Na–H]^+^ features increased the CCS relative to [M + H]^+^ by 2.5 ± 2.0 Å^2^, 4.7 ± 1.2 Å^2^, and 5.6 ± 1.4 Å^2^ in 43, 15, and 26 cases, respectively (± values indicate standard deviations throughout the manuscript). Sphingomyelin data is omitted from this analysis, as it exhibited significantly different behavior, with 15 cases of [M + H]^+^ all being larger than [M + Na]^+^ by 1.9 ± 1.1 Å^2^. This unusual trend of smaller ^DT^CCS_N2_ values for sodiated species compared to protonated species is likely related to the larger gas-phase conformations observed for sphingolipids, where the coordination of a metal cation does not add significantly to the ^DT^CCS_N2_. For negatively charged ions, [M + COOH]^−^ was observed to increase the CCS by 1.6 ± 1.9 Å^2^ over [M + Cl]^−^ for 26 cases; there were an insufficient number of cases in which lipid features were observed as both [M−H]^−^ and either [M + Cl]^−^ or [M + COOH]^−^ for statistical comparison.

### Quantitative mobility-mass correlations for a lipid atlas

**Fig. 4:**
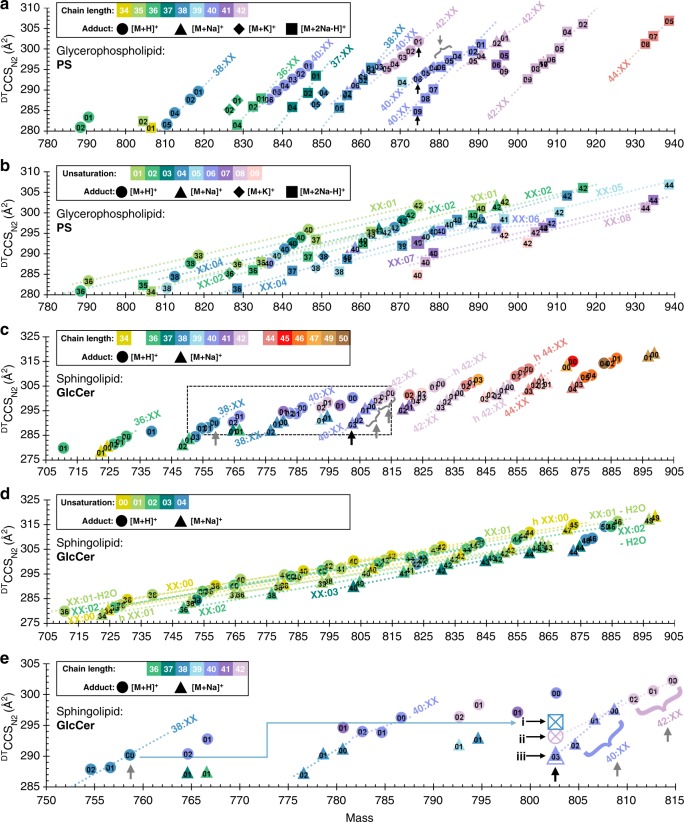
Quantitative correlations within the cation data for PS and GlcCer lipid classes. **a**, **b** ^DT^CCS_N2_ of PS lipids. For the same carbon chain length, PS lipids increase linearly in ^DT^CCS_N2_ with each loss of unsaturation (**a**). For the same degree of unsaturation, ^DT^CCS_N2_ values increase linearly with PS acyl chain length (**b**). **c**, **d** ^DT^CCS_N2_ of GlcCer lipids. GlcCer species exhibit longer chain lengths than PS, however, the same linear trends are observed for both number of double bonds (**c**) and for carbon chain length within the same double bond category (**d**). **e** A closer inspection of the boxed region highlighted in **c** demonstrates identification of the initially unknown lipid feature at 802.616 Da, 290.3 Å^2^ (denoted by a black arrow). Based on the quantitative ^DT^CCS_N2_ trends, the lipid feature is lower in ^DT^CCS_N2_ than the predictions for both GlcCer 38:00 + 2Na–H (295.8 Å^2^, indicated as (i)) and GlcCer 42:06 + H (293.1 Å^2^, indicated as (ii)). However, the unknown feature aligns well with the ^DT^CCS_N2_ predicted for GlcCer 40:03 + Na (290.1 Å^2^, indicated as (iii)), enabling high confidence identification of this lipid. Colors correspond to either summed chain length or degree of unsaturation, whereas shapes correspond to cation type, as specified in the corresponding panel legends. Numerical annotations within symbols correspond either to degree of unsaturation or carbon length, depending on the panel. Error bars represent standard errors for the ^DT^CCS_N2_ measurements and are all within the size of the markers. Specific lipid identifications, numerical measurement data, and number of measurements per data point (*n* = 8 or 16 repeat measurements for positive ionization mode data, *n* = 20, 21, or 28 repeat measurements for negative ionization mode data) are provided in Supplementary Data 1. Information for linear fits including slopes, R-squared values, and number of points per trend line are provided in Supplementary Data 2

Within each individual lipid class, highly linear mobility-mass correlations were observed (Fig. 4 and Supplementary Figs. 2–3). With either the degree of unsaturation or the chain length held constant, no secondary dependence on the modification type or adduct was found to influence these mobility-mass correlation trends. For example, the correlation of protonated PE lipids with a single double bond in the acyl chain is nearly identical to correlations observed for lipids with either a sodium adduct, hydroxylated beta carbon, or alternate number of double bonds. Thus, the empirical lipid trends are predominately a result of two primary structural features: the number of carbons in the acyl tail and the degree of unsaturation.

Within each ionization mode and for each lipid class, lipid features were grouped based on either the same number of double bonds or acyl chain carbons, and linear functions were fitted within each unique category to groups of three or more lipid features (Supplementary Data 2). Across the 7 lipid classes investigated, this yielded linear fits for 56 sets of lipid cations and 16 sets of lipid anions with varying degrees of unsaturation (Fig. 4a,  c, and Supplementary Figs. 2–3), and 44 sets of lipid cations and 10 sets of lipid anions with varying numbers of acyl chain carbons (Fig.4b, d, and Supplementary Figs. 2–3). In agreement with findings from Zhang et al., a linear equation was found to best describe the correlation between lipid CCS and mass^39^. Here, average *R*^2^ values of 0.98 (0.89 minimum) for common alkyl chain length and 0.99 (0.94 minimum) for common degree of unsaturation were observed for cation data; average *R*^2^ values of 0.98 (0.93 minimum) for common alkyl chain length and 1.00 (0.99 minimum) for common degree of unsaturation were observed for anion data. Although size is inherently expected to increase with mass, structural changes affect molecular density, and, in positive ionization mode, changes in the degree of unsaturation were found to be four times as influential on the gas-phase CCS as changes in alkyl chain length across all lipid classes (Supplementary Table 2) with slopes of 0.95 ± 0.16 Å^2^ per mass unit and 0.23 ± 0.03 Å^2^ per mass unit, respectively. In negative ionization mode, changes in the degree of unsaturation were found to be three times as influential on CCS as changes in alkyl chain length (Supplementary Table 3) with slopes of 0.62 ± 0.11 Å^2^ per mass unit and 0.20 ± 0.02 Å^2^ per mass unit, respectively.

The larger deviation in slopes observed for lipids with a common degree of unsaturation is attributed to a combination of mass overlap and poorly resolved drift times. Lipids of common chain length tend to occur with multiple degrees of unsaturation, and the loss of one double bond (equivalent to an addition of 2 hydrogen atoms, or 2.02 Da) overlaps the third isotope of the smaller, more unsaturated lipid in mass, decreasing the measured CCS of the heavier species, which would require greater than 40,000 mass resolving power to isolate. In addition to isotopic interference, mass overlap also occurs for lipids of varied structure and adduct, with measurements reflecting a weighted average of all isomers of varied acyl chain length, double bond positions, sn-locations, etc. However, in accordance with our observations and those of other studies, the magnitude of influence of these isomers is negligible at the current instrument mobility resolution (ca. 60)^40^ and compared to the influence of the variables that are the focus of this work (i.e. mass, chain length, degree of unsaturation, etc.)^29^. For example, at nominal mass 874 corresponding to PS 40:09 + 2Na–H, PS 40:06 + K, and PS 42:01 + H (Fig. 4a; 874.46, 874.50, and 874.65, respectively), the latter nominal mass isomer, PS 42:01 + H, has a larger ^DT^CCS_N2_ and is also more abundant in the spectra, which results in PS 40:06 + K to be calculated at a CCS higher than predicted from trends in the data. In another example, PS 42:09 + Na, PS 42:08 + Na, and PS 42:07 + Na have CCS values which are higher than expected (Fig. 4a) and which is attributed to spectral overlap with PS 40:03 + K, PS 40:02 + K, and PS 40:01 + K, respectively, although these potassium adducted species were not able to be resolved as unique features. In a third example, shown in Supplementary Fig. 4, the fragmentation data of PE 36:02 [M−H]^−^ indicates the presence of both PE 18:1/18:1 and PE 18:0/18:2 (and/or PE 18:2/18:0) isomers. We present these examples to demonstrate the level of complexity which is present even for class-purified lipid standards.

### Identification by CCS

The quantitative relationships developed here to describe groups of lipid features differing only in either chain length, degree of unsaturation, or adduct type can be used to identify lipid features that are ambiguous to characterize by mass alone. For example, one GlcCer feature observed in this study could be assigned three probable mass-identifications within 5 ppm mass error. This unknown lipid is indicated by the black arrow in Fig. 4c (802.62 Da, 290.3 Å^2^) and, based on mass measurement, could be identified as either (1) GlcCer 38:00 + 2Na–H (+1 ppm), (2) GlcCer 42:06 + H (−5 ppm), or (3) GlcCer 40:03 + Na (−2 ppm). To reduce this ambiguity, linear and differential trends defined by IM-MS in this work were used to predict the ^DT^CCS_N2_ of the three possible identifications, and the predicted values were compared to the empirically measured cross section of the unknown feature to make a high-confidence identification. The CCS of the first possibility can be predicted based on the previously defined differential relationship between adduct types. Because GlcCer 38:00 was observed as [M + H]^+^, the CCS of the same lipid as [M + 2Na–H]^+^ can be predicted ~ 5.6 Å^2^ higher, at 295.8 Å^2^. The CCS of the second possible identification, protonated GlcCer 42:06, was predicted at 293.1 Å^2^ from its linear relation to other [M + H]^+^ 42-carbon GlcCer lipids observed with lower degrees of unsaturation. The last probable mass-identification, sodiated GlcCer 40:03, was predicted at 290.1 Å^2^, in close agreement with the measured value, from its linear relation with other [M + Na]^+^ 40-carbon GlcCer lipids observed with lower degrees of unsaturation. As depicted in Fig. 4e, because the CCS of the first and second possibilities were significantly larger (1.9% and 1.0%, based on difference from the measured value) and the third possibility aligned well with the measured value (0.1%), the identification of the unknown feature was accepted with high confidence as the [M + Na]^+^ form of GlcCer 40:03.

### Lipid mixture analysis

**Fig. 5:**
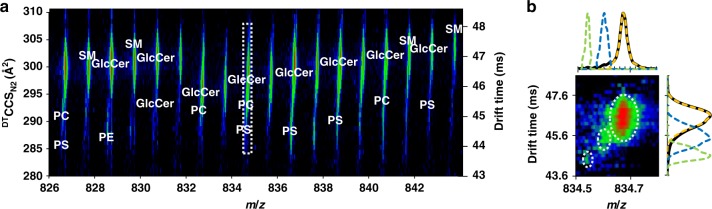
Spectrum of a mixture of PE, PS, PC, SM, and GlcCer lipids. Data was obtained in positive ionization mode. **a** A 2D IM-MS spectrum for a mixture of lipid extracts, with individual lipid classes annotated. **b** Selected region demonstrated multiple lipid features residing within a narrow mass and mobility range. Dotted lines represent extracted mobility and mass spectra for circled features, which include GlcCer (orange), PC (blue), and PS (green) lipids

Figure 5 contains the 2D IM-MS spectrum of a mixture of PE, PS, PC, SM, and GlcCer lipids. Identifications for the mixture were made by matching to known lipid features in the individually analyzed lipid extracts. While high resolution mass measurement is often enough to resolve and confidently identify lipids by accurate mass, in cases where significant feature overlap is observed, i.e. in complex samples with multiple lipid classes at varying concentrations, mass information alone is insufficient (Supplementary Figs. 2–3, 5). For example, three features are found at a nominal mass of 834 Da within a 0.3 Da window (Fig. 5b) and correspond to the sodiated forms of GlcCer 42:01, PC 38:03, and PS 38:04, and a more complex sample could also contain numerous other lipid species within a ± 0.1 Da mass window (e.g. sodiated PE O-O-38:03; protonated PC 40:06, PE 43:06, PS 39:00, PS O-40:00, or lactosylceramide h 32:01; potassiated GlcCer h 41:02; [M + 2Na–H]^+^ PC 36:00, PC O-37:00, PE 39:00, or PE O-40:00; based on a non-exhaustive database search) of the most abundant peak (sodiated GlcCer 42:01), in addition to mass overlap with isotopes of lipids with higher degrees of unsaturation observed in the complex spectra. The two phospholipids are present in very low relative abundance, which results in their peak features being challenging to resolve in either the IM or MS dimension alone, with the integrated spectrum (black line) for each dimension exhibiting only a single distribution. With the combined IM-MS information, however, three features are readily observed and can be extracted to resolve each of the three lipids in both IM and MS space. This in turn allows accurate mass and ^DT^CCS_N2_ information to be obtained. Using the CCS information, more confident identifications of the components in this narrow mass window can be made by correlating the two lower CCS features to PS and PC lipids, and the high CCS feature to GlcCer.

## Discussion

In this work, 456 ^DT^CCS_N2_ measurements are presented for 4 classes of glycerophospholipids (PA, PC, PE, PS) and 3 classes of sphingolipids (Cer, SM, GlcCer). In complex IM-MS spectra, lipids can be observed in a region separate from other biomolecular classes and discerned within this region by lipid category, trending uniquely relative to head group size. Multiple adducts of each lipid species were observed in both positive ionization mode and, to a lesser degree, negative ionization mode, with relative ^DT^CCS_N2_ values being generally commensurate with the relative size of the adducts, that is, [M + 2Na–H]^+^ > [M + K]^+^ > [M + Na]^+^ > [M + H]^+^ and [M + COOH]^−^ > [M + Cl]^−^. For lipids of common head group, changes in the degree of unsaturation were found to be four times as influential on conformational broadening as were changes in alkyl chain length. We anticipate that the quantitative trends determined from this data and their generalizability to other lipid classes will aid in identifications of lipid features in future lipidomics analyses, when mass information alone is found to be insufficient. The conformational lipid atlas presented here represents the most precise and comprehensive structural analysis of lipids to date and should both stimulate and facilitate bioinformatic efforts in using ion mobility-derived CCS measurements in quantitative lipidomics research.

## Methods

### Preparation of lipid samples

HPLC grade solvents and buffers were obtained from Sigma-Aldrich (St. Louis, MO, USA). Lipid standards including phosphatidylethanolamine (PE, chicken egg), phosphatidylcholine (PC, chicken egg), phosphatidylserine (PS, porcine brain), sphingomyelin (SM, porcine brain), and cerebroside (GlcCer, porcine brain), were purchased as purified TLC fractions from Avanti Polar Lipids (Birmingham, AL, USA) and the dry extracts were reconstituted in chloroform prior to analysis. Lipid standards were diluted in 33% chloroform/67% methanol by volume to a final concentration of 10 μg/mL for analysis. Identification of lipids was based on exact mass measurements and the Lipid Metabolites and Pathways Strategy (LIPID MAPS) Structural Database (LMSD) and the Scripps Center for Metabolomics Metabolite Database (METLIN).

### Instrumentation

Three independent high resolution drift tube IM-MS instruments (IM-QTOF 6560, Agilent Technologies) were utilized to acquire accurate mass and CCS measurements from lipid samples^28,40^. The instrument consists of an orthogonal electrospray ionization (ESI) source (Agilent Jet Stream) with a heated sheath gas nebulizer for desolvation and focusing of ions at atmospheric pressure. A single bore, resistively coated, glass capillary is used to transfer ions into the vacuum system. As ions exit the transfer capillary, they are directed by a high pressure ion funnel to a second ion funnel with two wire grids for ion trapping and gating. Ions accumulate in the trapping funnel and are pulsed into the uniform field IM drift tube which is approximately 78 cm in length. An additional rear ion funnel refocuses ions as they exit the drift tube and pass to a lower pressure region via a hexapole ion guide. Ions pass through a quadrupole mass filter and collision cell before mass measurement is performed in an orthogonal time-of-flight mass spectrometer.

### Experimental parameters

All 2D IM-MS spectra were obtained via either direct infusion at a flow rate of 10 μL/min or automated flow injection^41^ at 50 μL/min in positive and negative mode ESI. For direct infusion methods, a syringe pump was coupled to the ESI source. For flow injection methods, a liquid chromatography system (1290, Agilent Technologies) with no chromatography column was coupled to the ESI source and operated with a 100 µL sample loop. Furthermore, in flow injection experiments, analyte was injected (10 µL in positive mode, 20 µL in negative mode) at a flow rate of 800 µL/min (solvent system was 20% water/80% acetonitrile by volume), which was decreased to 50 µL/min after 0.15 min, resulting in stable analyte signal for about 3.5 min, and then increased to 800 µL/min after 4 min to purge any remaining analyte. Nitrogen sheath gas at 12 L/min and 600–700 K and nitrogen drying gas at 10 L/min and 540 K were used in the Agilent Jet Stream ion source. The ion source potential emitter was held at ground voltage, the capillary entrance was biased to ± 4.5 kV, and the nozzle was biased to ± 1.8 kV, depending on the ion polarity. The high-pressure ion funnel was operated at ca. 4.8 Torr with RF 200 V_pp_ at 1.5 MHz and 150 V DC, the trapping ion funnel was operated at 3.8 Torr with RF 200 V_pp_ at 1.2 MHz and 180 V DC, and the rear funnel was operated at ca. 4.0 Torr with RF 180 V_pp_ at 1.2 MHz and 200 V DC. The IM was pressurized to ca. 4.0 Torr and ca. 300 K with ultrahigh purity nitrogen, and the drift potential was varied between 850 to 1450 V (*E/N* range of 7 to 15 Td) in increments of 100 V, dwelling at each drift potential for 30 s, similar to a standardized stepped-field method^42^. Data was acquired and processed using modified MassHunter software (Data Acquisition and IM-MS Browser, Agilent Technologies).

### Calibration methods

Mobility and mass calibration was applied externally using homogenously substituted fluoroalkyl phosphazenes (Agilent tune mix, ca. 100 to 3000 mass). In addition, tetraalkylammonium (TAA) salts, which fall outside the IM and MS range of lipids, were added to all samples as internal standards for positive mode analysis. Results obtained without the TAA calibrants were similar, indicating that the presence of these cations did not significantly suppress the lipid ion signals. TAA salts of 98% purity or greater and varying alkyl chain lengths were obtained from several sources: TAA4, TAA6, TAA7, TAA10, TAA12, and TAA16 were purchased from Sigma Aldrich, TAA3, TAA5, and TAA8 were purchased from Acros Organics (Morris Plains, New Jersey, USA), and TAA18 was purchased from Alfa Aesar (Ward Hill, MA, USA). TAA3 to TAA8 were prepared in 50% methanol/50% water. TAA10, TAA12, TAA16, and TAA18 were prepared in 50% methanol/50% isopropanol. Final concentrations for analyses were ca. 1 μg/mL.

### Prediction of CCS

Drift tube CCS values in nitrogen gas, ^DT^CCS_N2_, were empirically determined via the Mason-Schamp relationship, using a standardized stepped electric field method. Various instrument settings, including ranges of drift fields and ion optical settings, have been evaluated in an interlaboratory study in order to optimize measurement parameters necessary to obtain ^DT^CCS_N2_ measurements that were reproducible to within 0.5%^42^. While ion optical parameters were observed to affect the CCS measurement, prior work established that these relative shifts in drift times are related to fringing electric fields as opposed to ion heating effects which influence peak shapes^42,43^. Mobility-mass correlations were performed with simple linear regression analyses. Lipid features separated by class, modification, and adduct type were grouped either by those sharing the number of double bonds or by those of similar acyl chain length. Linear functions were fitted within each unique category to groups of three or more lipid features (average number of features is reported as number of points per line in Supplementary Table 2). With either the degree of unsaturation or the chain length held constant, no secondary dependence on lipid modification type or adduct was found to influence these mobility-mass correlation trends, permitting averaging of calculated slopes across these qualifiers. For each lipid class and an average across all classes described in this work, Supplementary Table 2 lists the average and percent relative standard deviation of mobility-mass correlation slopes, average and minimum coefficients of determination describing the data for the individual trend lines, the number of trend lines per class, and the average number of data points included in each trend line.

Adduct correlations were performed with a simple differential analysis. ^DT^CCS_N2_ values of mass-identified lipid features differing only in adduct type were compared, and this data is summarized in Supplementary Fig. 1. Influence of cation form on ^DT^CCS_N2_ was found to be independent of lipid class except for sphingolipids, as described in the results, thus adduct pairings with three or more occurrences were averaged while omitting the sphingolipids.

Prediction of ^DT^CCS_N2_ for lipids can be performed from the mass of the ionic form and the quantitative relationships developed here, which describe empirical structural changes based on multiple pieces of analytical information. Cross-section values for lipid features that have not previously been reported can be extrapolated or interpolated from the linear equations of mobility-mass correlations described here. Comparatively, cross sections of unique lipids that have been reported in one or more cation forms can be used to calculate cross sections of the same lipids with alternate cation forms. Here we use accurate mass measurement (10 ppm threshold) as the primary identifier, but for features with multiple possible mass identifications, the developed predictive capabilities are utilized in order to evaluate the possibilities and determine the appropriate lipid identification.

### Reporting summary

Further information on experimental design is available in the Nature Research Reporting Summary linked to this article.

## Supplementary information

Supplementary Information

Peer Review File

Description of Additional Supplementary Files

Supplementary Data 1

Supplementary Data 2

Reporting Summary

## Data Availability

A table of information and measurements for the lipids investigated in this study is provided in Supplementary Data 1. ^DT^CCS_N2_ data are also available from an online resource, the Unified CCS Compendium (https://mcleanresearchgroup.shinyapps.io/CCS-Compendium/)^36^. A reporting summary for this Article is available as a Supplementary Information file. All other data supporting the findings of this study are available from the corresponding author upon request.
