# Supplementary material for: Ion mobility conformational lipid atlas for high confidence lipidomics
Source: Nat Commun. 2019 Feb 28;10:985. doi: 10.1038/s41467-019-08897-5 (PMC6395675; doi:10.1038/s41467-019-08897-5)
Supplement: Supplementary file 1 — Supplementary Information [file 41467_2019_8897_MOESM1_ESM.pdf]

Supplementary Information

Ion Mobility Conformational Lipid Atlas for High Confidence Lipidomics

Leaptrot, et al.

Ion Mobility Conformational Lipid Atlas for High Confidence Lipidomics

Katrina L. Leaptrot[1], Jody C. May[1], James N. Dodds[1], John A. McLean[1,*]

[1]Center for Innovative Technology, Department of Chemistry, Vanderbilt Institute of Chemical Biology, Vanderbilt Institute for Integrative Biosystems Research and Education, Vanderbilt-Ingram Cancer Center, Vanderbilt University, Nashville, Tennessee 37235, United States

*To whom correspondence should be addressed. E-mail: john.a.mclean@vanderbilt.edu

Contents:

Supplementary Figure 1 – Histograms of adduct effect on collision cross section magnitude.

Supplementary Figure 2 – $^{DT}CCS_{N2}$ vs. mass plots with linear fits to observed structural trends for positively charged ions.

Supplementary Figure 3 – $^{DT}CCS_{N2}$ vs. mass plots with linear fits to observed structural trends for negatively charged ions.

Supplementary Figure 4 – Fragmentation spectra of PE 36:02 anion.

Supplementary Figure 5 – Ion mobility spectra for one set of lipid isomers.

Supplementary Table 1 – Linear regression data for lines graphed in Fig. 2.

Supplementary Table 2 – Variables for linear fits to positive ionization mode data depicted in Fig. 4 and Supplementary Fig. 2.

Supplementary Table 3 – Variables for linear fits to negative ionization mode data depicted in Supplementary Fig. 3.

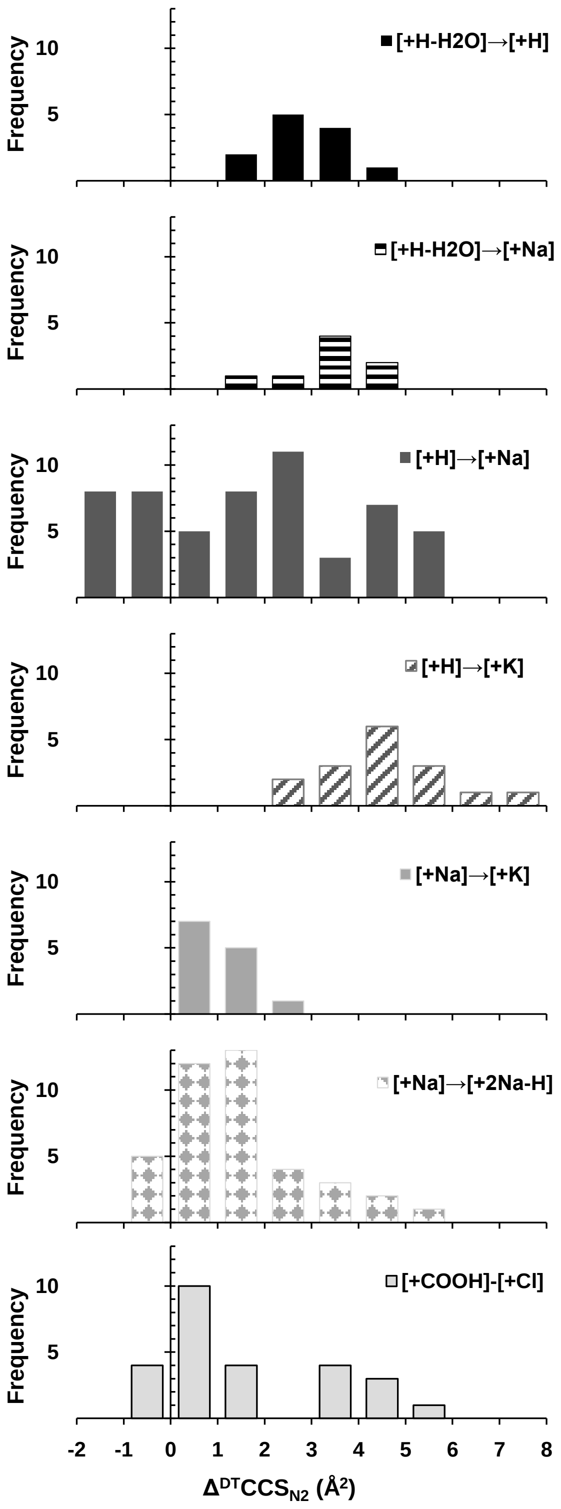

Supplementary Fig. 1. Histograms summarizing observed change in collision cross section ($\Delta\ ^{DT}CCS_{N2}$) across the various identified adducts. The CCS for unique lipid molecules observed with multiple charge carriers were compared. For example, in the bottom graph, there were 26 occurrences of the same lipid observed as both $[M+Cl]^-$ and $[M+COOH]^-$, and in 10 of those occurrences, the $[M+COOH]^-$ had a CCS greater than that of the $[M+Cl]^-$ by 0 to 1 Å^2.

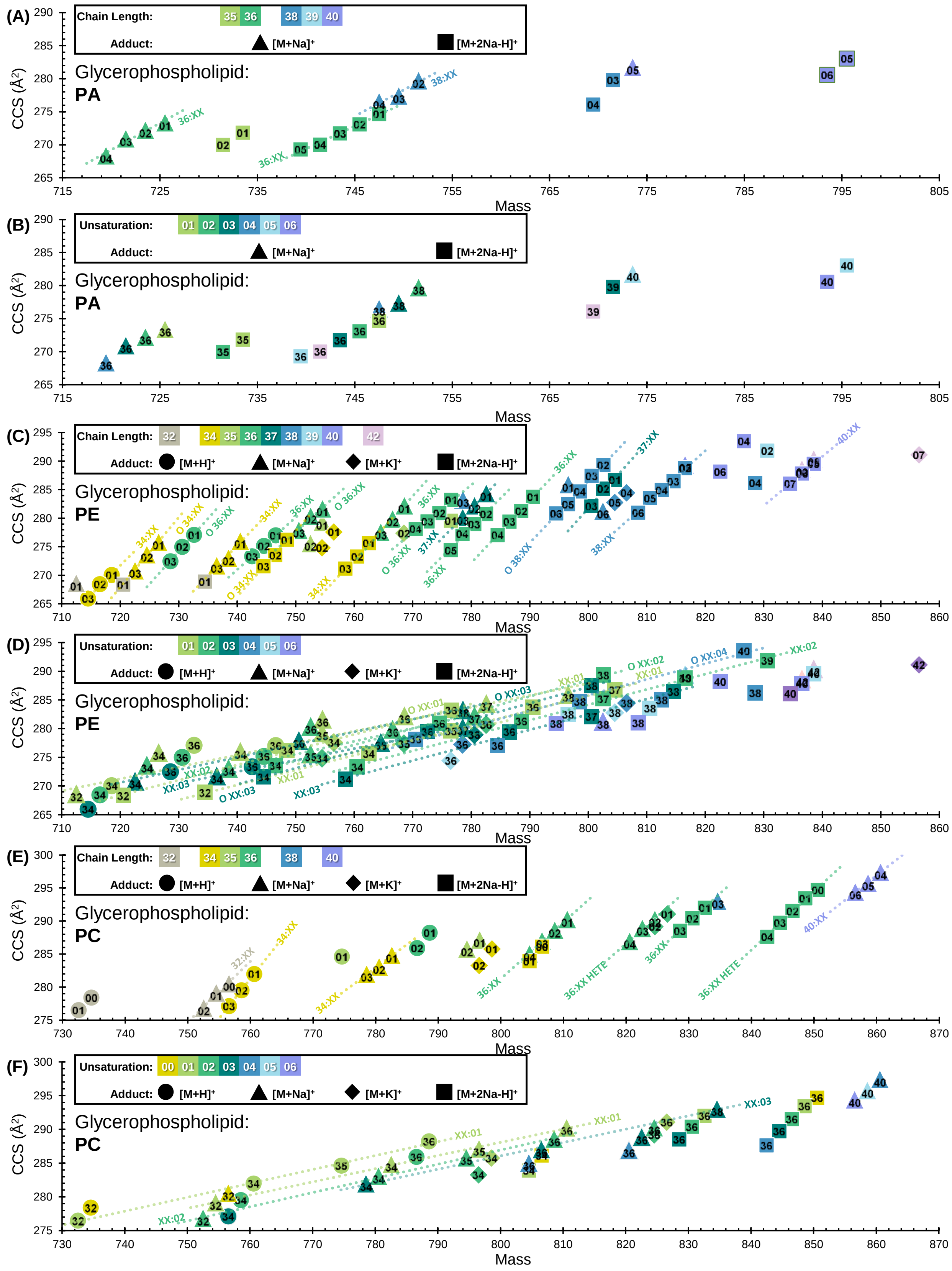
(A)
Chain Length: 35 36 38 39 40
Adduct: [M+Na]+ [M+2Na-H]+
Glycerophospholipid:
PA
CCS (Å²)
Mass
(B)
Unsaturation: 01 02 03 04 05 06
Adduct: [M+Na]+ [M+2Na-H]+
Glycerophospholipid:
PA
CCS (Å²)
Mass
(C)
Chain Length: 32 34 35 36 37 38 39 40 42
Adduct: [M+H]+ [M+Na]+ [M+K]+ [M+2Na-H]+
Glycerophospholipid:
PE
CCS (Å²)
Mass
(D)
Unsaturation: 01 02 03 04 05 06
Adduct: [M+H]+ [M+Na]+ [M+K]+ [M+2Na-H]+
Glycerophospholipid:
PE
CCS (Å²)
Mass
(E)
Chain Length: 32 34 35 36 38 40
Adduct: [M+H]+ [M+Na]+ [M+K]+ [M+2Na-H]+
Glycerophospholipid:
PC
CCS (Å²)
Mass
(F)
Unsaturation: 00 01 02 03 04 05 06
Adduct: [M+H]+ [M+Na]+ [M+K]+ [M+2Na-H]+
Glycerophospholipid:
PC
CCS (Å²)
Mass

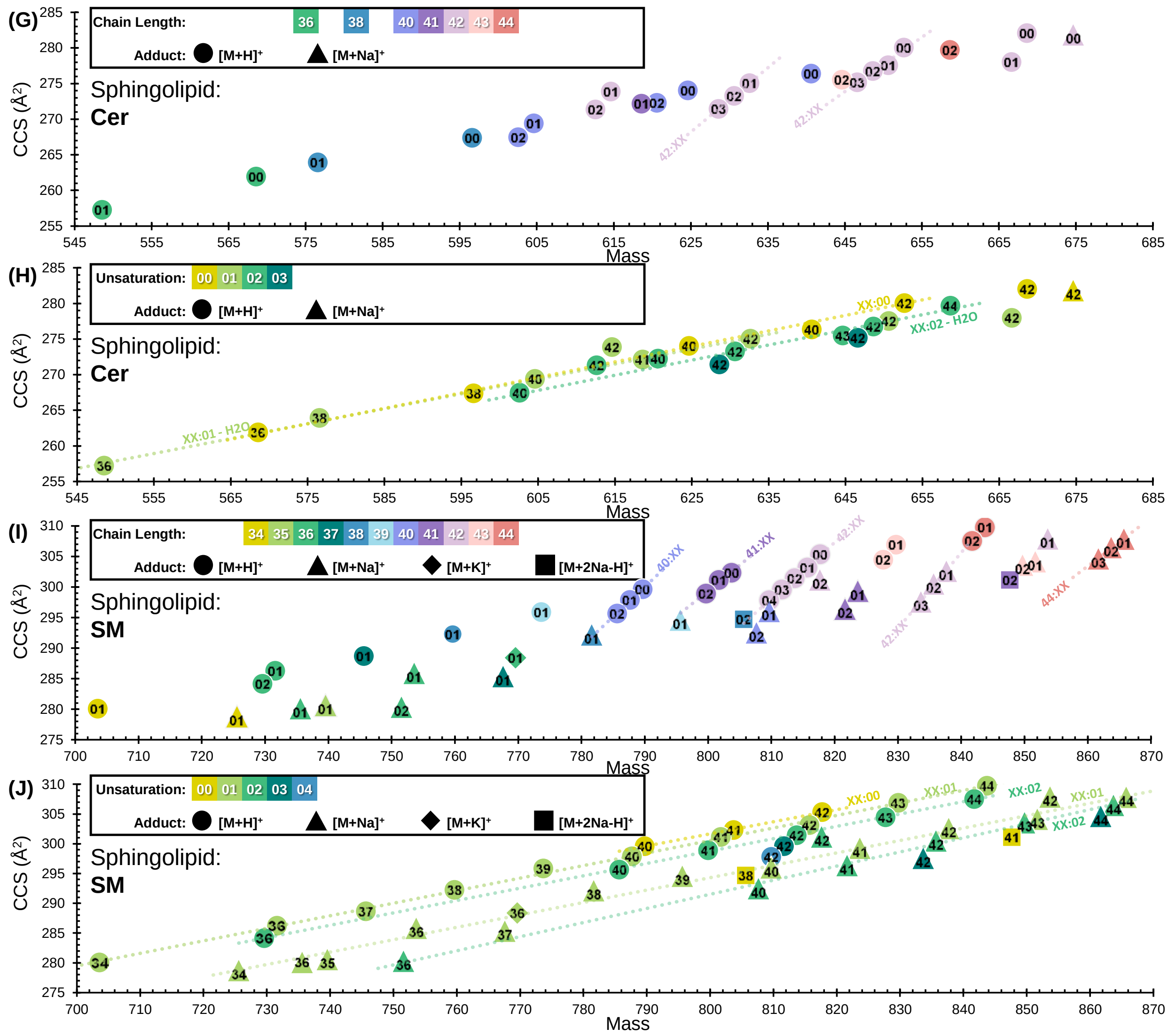

Supplementary Fig. 2. Plots of quantitative correlations within lipid classes of positive ionization mode. Colors correspond to either summed chain length or degree of unsaturation and shapes correspond to cation type, as specified in the legend. Numbers within the symbols correspond to either the degree of unsaturation, or the number of acyl chain carbons. Error bars are within the size of the markers. (A) Glycerophosphatidic acid (PA) chain length trends, and (B) PA unsaturation trends. (C) Glycerophosphoethanolamine (PE) chain length trends, and (D) PE unsaturation trends. (E) Glycerophosphotidylcholine (PC) chain length trends, and (F) PC unsaturation trends. (G) Ceramide (GlcCer) chain length trends, and (H) GlcCer unsaturation trends. (I) Sphingomyelin (SM) chain length trends, and (J) SM unsaturation trends.

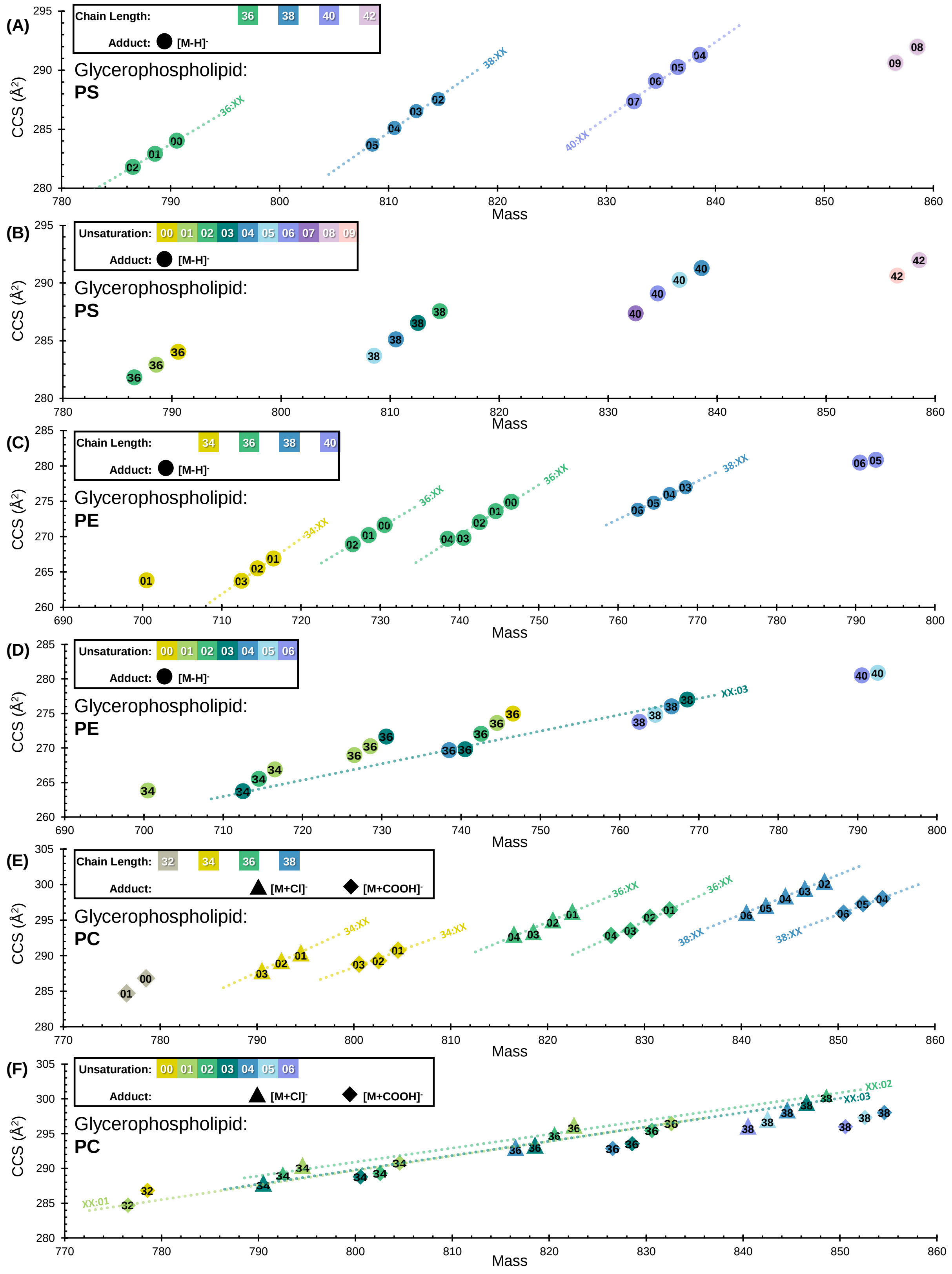
(A)
Chain Length: 36 38 40 42
Adduct: [M-H]-
Glycerophospholipid:
PS
CCS (Å2)
Mass
(B)
Unsaturation: 00 01 02 03 04 05 06 07 08 09
Adduct: [M-H]-
Glycerophospholipid:
PS
(C)
Chain Length: 34 36 38 40
Adduct: [M-H]-
Glycerophospholipid:
PE
(D)
Unsaturation: 00 01 02 03 04 05 06
Adduct: [M-H]-
Glycerophospholipid:
PE
(E)
Chain Length: 32 34 36 38
Adduct: [M+Cl]- [M+COOH]-
Glycerophospholipid:
PC
(F)
Unsaturation: 00 01 02 03 04 05 06
Adduct: [M+Cl]- [M+COOH]-
Glycerophospholipid:
PC

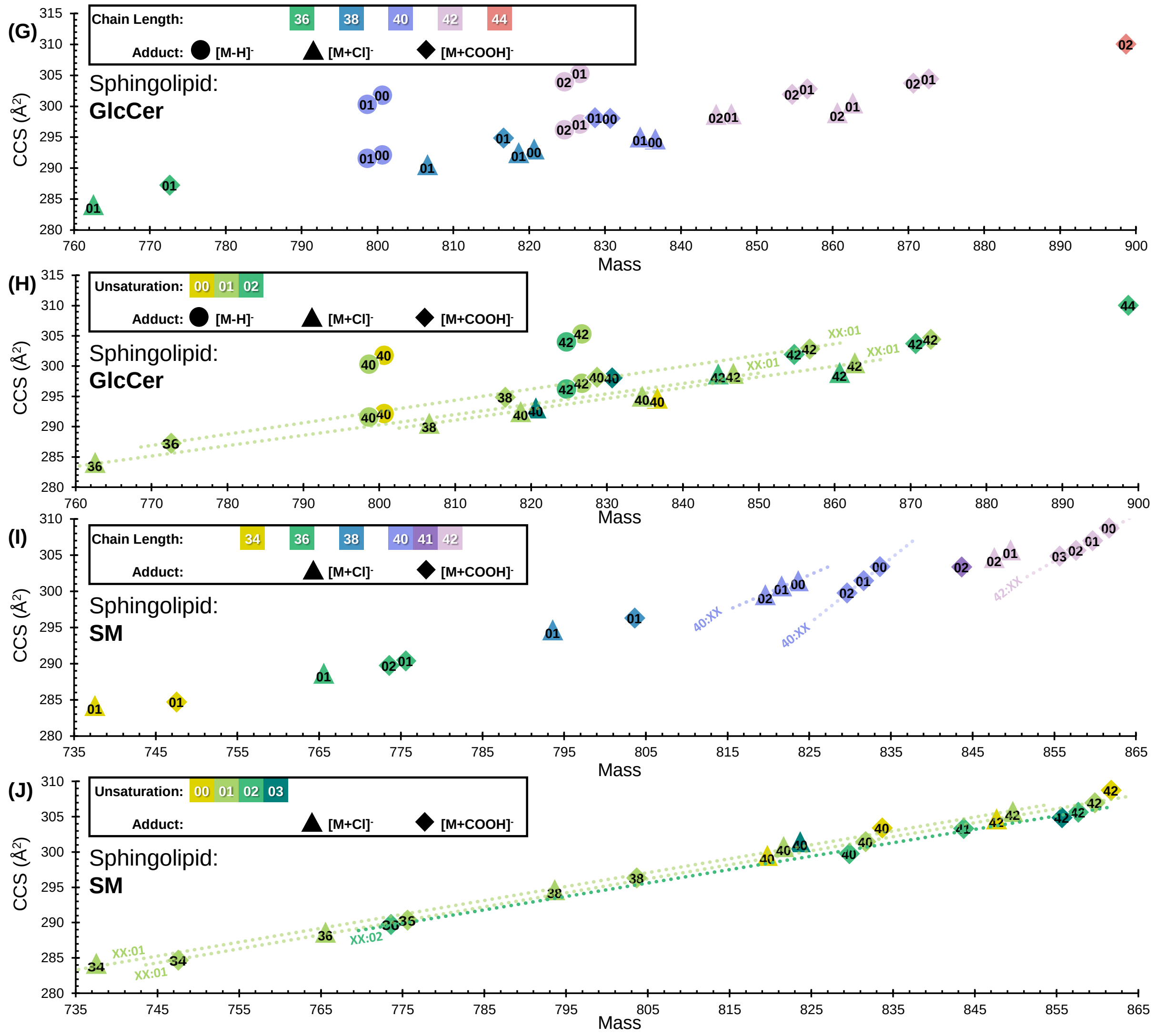

Supplementary Fig. 3. Plots of quantitative correlations within lipid classes of negative ionization mode. Colors correspond to either summed chain length or degree of unsaturation and shapes correspond to anion type, as specified in the legend. Numbers within the symbols correspond to either the degree of unsaturation, or the number of acyl chain carbons. Error bars are within the size of the markers. (A) Glycerophosphatidylserine (PS) chain length trends, and (B) PS unsaturation trends. (C) Glycerophosphoethanolamine (PE) chain length trends, and (D) PE unsaturation trends. (E) Glycerophosphotidylcholine (PC) chain length trends, and (F) PC unsaturation trends. (G) Ceramide (GlcCer) chain length trends, and (H) GlcCer unsaturation trends. (I) Sphingomyelin (SM) chain length trends, and (J) SM unsaturation trends.

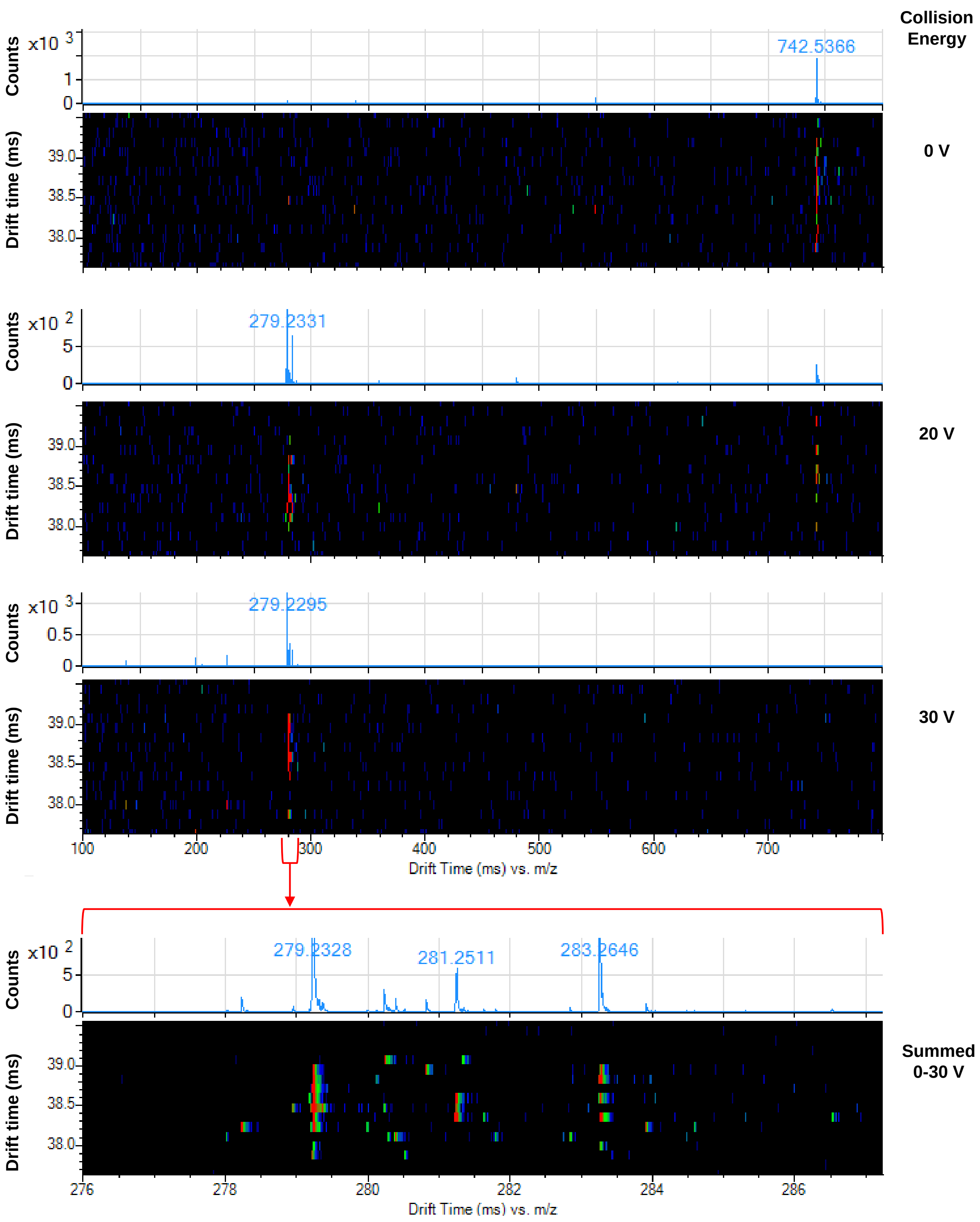

Supplementary Fig. 4. Fragmentation spectra for PE *m/z* 742 in negative ionization mode. Fragments at *m/z* 279, 281, and 283 represent alkyl chain ions dissociated at the sn1 and sn2 positions, and their appearance indicates a mixture of alkyl chain lengths exist for the identified PE 36:02 $[M\text{-}H]^-$ peak at *m/z* 742. The *m/z* 281 fragment represents an 18:01 fatty acid, indicating the presence of PE (18:1/18:1). The *m/z* 279 and 283 fragments represent an 18:00 and 18:02 fatty acid, respectively, indicating the presence of PE (18:0/18:2) and/or PE (18:2/18:0).

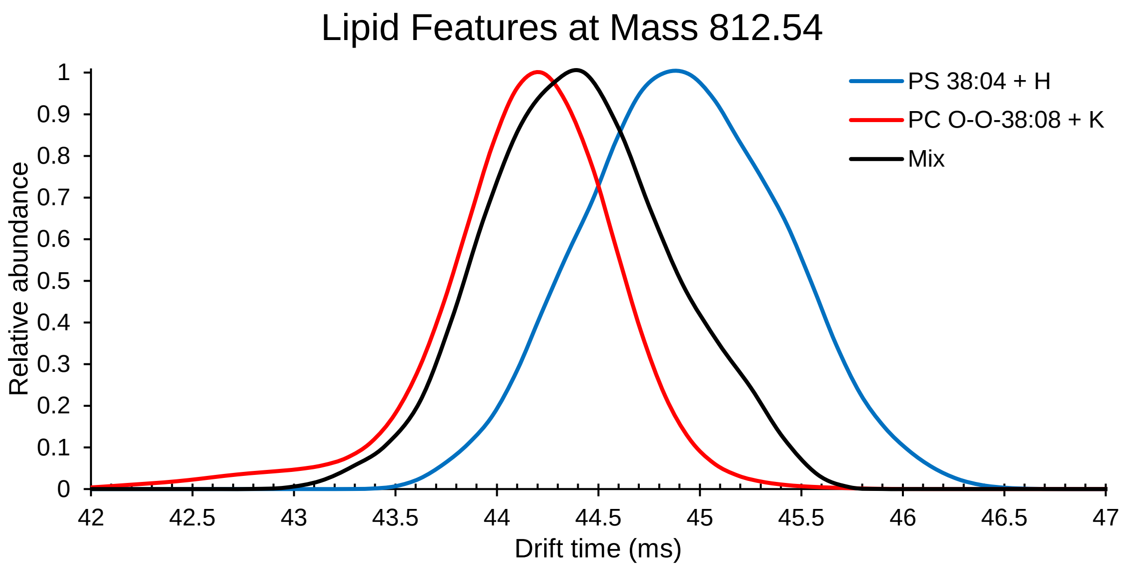

Supplementary Fig. 5. Plot of individual drift times for lipid features found at 812.54 Da. These two lipid ions would require ca. 100,000 mass resolution to be resolved at half-height in the mass spectrum. The PS 38:04 + H (blue trace) and PC 38:08 + K (red trace) IM spectra are obtained from the IM-MS analysis of individual standards, whereas the IM distribution for the mixture (black trace) was obtained from a mixture of the PS and PC standards. Though the individual ions can be clearly differentiated by IM, the mixture is unresolved, underscoring the need for increased IM resolving power in lipidomic studies.

Supplementary Table 1. Summary of information for trend lines depicted in Fig. 2. "N (Points in trend line)" indicates the number of unique lipid data points (*m/z*, $^{DT}CCS_{N2}$) included in each trend line; to be grouped into a trend line, lipids must be of the same lipid category.

Lipid Category	N (points in trend line)	Slope	Intercept	R^2
Phospholipids	127	0.1452	180.10	0.9168
Sphingolipids	329	0.1657	153.97	0.6660

Supplementary Table 2. Tables summarizing statistics for positive ionization mode linear fits to $^{DT}CCS_{N2}$ vs. mass data. (A) Statistics relating to lipids common in modification, adduct, and alkyl chain length, but differing in degree of unsaturation, arranged by head group. (B) Statistics relating to lipids common in modification, adduct, and degree of unsaturation, but differing in length of alkyl chains, arranged by head group.

Lipids in Trend Share Number of Carbons (Cations)

(A)

	Slope (Å^2 per *m/z*)		R^2		Number	
	**Avg**	**%RSD**	**Avg**	**Min**	**Trendlines**	**Points/line**
**PA**	0.76	9%	0.961	0.938	3	4.0
**PE**	1.04	11%	0.988	0.953	17	3.4
**PC**	0.88	17%	0.993	0.981	8	3.4
**PS**	0.92	18%	0.978	0.897	12	4.6
**SM**	0.96	18%	0.989	0.968	5	3.4
**GlcCer**	0.93	18%	0.966	0.886	9	3.4
**Cer**	0.83	11%	0.979	0.958	2	3.5
	**Avg**	**Avg**	**Avg**	**Min**	**Sum**	**Avg**
**Summary**	0.95	17%	0.981	0.886	56	3.7

Lipids in Trend Share Degree of Unsaturation (Cations)

(B)

	Slope (Å^2 per *m/z*)		R^2		Number	
	**Avg**	**%RSD**	**Avg**	**Min**	**Trendlines**	**Points/line**
**PA**	-	-	-	-	0	-
**PE**	0.25	13%	0.994	0.978	12	3.8
**PC**	0.20	3%	0.998	0.996	4	3.8
**PS**	0.24	7%	0.984	0.941	10	4.1
**SM**	0.21	6%	0.996	0.986	5	7.2
**GlcCer**	0.21	6%	0.998	0.994	11	5.3
**Cer**	0.21	2%	0.996	0.990	3	4.3
	**Avg**	**Avg**	**Avg**	**Min**	**Sum**	**Avg**
**Summary**	0.23	11%	0.993	0.941	44	4.7

Supplementary Table 3. Tables summarizing statistics for negative ionization mode linear fits to $^{DT}CCS_{N2}$ vs. mass data. (A) Statistics relating to lipids common in modification, adduct, and alkyl chain length, but differing in degree of unsaturation, arranged by head group. (B) Statistics relating to lipids common in modification, adduct, and degree of unsaturation, but differing in length of alkyl chains, arranged by head group

Lipids in Trend Share Number of Carbons (Anions)

(A)

	Slope (Å^2 per *m/z*)		R^2		Number	
	**Avg**	**%RSD**	**Avg**	**Min**	**Trendlines**	**Points/line**
**PA**	-	-	-	-	0	-
**PE**	0.67	15%	0.987	0.958	4	3.8
**PC**	0.56	10%	0.968	0.927	6	3.7
**PS**	0.61	9%	0.993	0.985	3	3.7
**SM**	0.68	31%	0.982	0.971	3	3.3
**GlcCer**	-	-	-	-	0	-
**Cer**	-	-	-	-	0	-
	**Avg**	**Avg**	**Avg**	**Min**	**Sum**	**Avg**
**Summary**	0.62	18%	0.980	0.927	16	3.6

Lipids in Trend Share Degree of Unsaturation (Anions)

(B)

	Slope (Å^2 per *m/z*)		R^2		Number	
	**Avg**	**%RSD**	**Avg**	**Min**	**Trendlines**	**Points/line**
**PA**	-	-	-	-	0	-
**PE**	0.24	-	0.998	0.998	1	3.0
**PC**	0.20	3%	0.999	0.999	3	3.0
**PS**	-	-	-	-	0	-
**SM**	0.20	2%	0.998	0.997	3	4.7
**GlcCer**	0.18	4%	0.995	0.988	3	3.0
**Cer**	-	-	-	-	0	-
	**Avg**	**Avg**	**Avg**	**Min**	**Sum**	**Avg**
**Summary**	0.20	9%	0.997	0.988	10	3.5
