# Supplementary material for: Ion mobility conformational lipid atlas for high confidence lipidomics
Source: Nat Commun. 2019 Feb 28;10:985. doi: 10.1038/s41467-019-08897-5 (PMC6395675; doi:10.1038/s41467-019-08897-5)
Supplement: Supplementary file 3 — Description of Additional Supplementary Files [file 41467_2019_8897_MOESM3_ESM.docx]

**Description of Supplementary Files**

**File Name:** Supplementary Data 1

**Description:** Collision cross section data for all lipids investigated. “ID” indicates lipid identification and “Modification” indicates lipid modification with nomenclature as described in the manuscript text. “Measured m/z” and “Mass Accuracy (ppm)” indicate the measured m/z and mass accuracy, respectively. “K0” and “K0 SD” indicate the reduced mobility and its standard deviation, respectively. “CCS,” “CCS SD,” and “RSD” indicate the DTCCSN2, its standard deviation, and its relative standard deviation, respectively. ”N” indicates the number of repeat measurements for each entry.

**File Name:** Supplementary Data 2

**Description:** Table summarizing information for trend lines shown in Figure 4, Supplementary Figure 2, and Supplementary Figure 3. “Lipid Modification” indicates lipid modification with nomenclature as described in the manuscript text. “N (Points in trend line)” indicates the number of unique lipid data points (m/z, DTCCSN2) included in each trend line; to be grouped into a trend line, lipids must share class, adduct type, modification, and either alkyl chain length or degree of unsaturation. Only groups of three or more are included. All features came from analytical standard total extracts from Avanti Polar Lipids.
