# Supplementary material for: Ion mobility conformational lipid atlas for high confidence lipidomics
Source: Nat Commun. 2019 Feb 28;10:985. doi: 10.1038/s41467-019-08897-5 (PMC6395675; doi:10.1038/s41467-019-08897-5)
Supplement: Supplementary file 2 — Peer Review File [file 41467_2019_8897_MOESM2_ESM.pdf]

Reviewers' comments:

Reviewer #1 (Remarks to the Author):

In this manuscript, the authors compiled a structural database of 354 mass-resolved collision cross section (CCS) values within sphingolipid and glycerophospholipid categories using IM-MS in positive ionization mode. The authors then used this information to analyze the primary differences between lipid categories. In their assays, acyl tail length and degree of unsaturation were found to be the primary structural descriptors for the different lipid categories and classes. The data included in this manuscript is novel as no group to date has done such an extensive characterization of lipid structures with IM-MS. However, I feel that the data from negative ionization mode also needs to be included for the manuscript to fully influence lipidomic analyses. Many of the lipids studied (i.e. phosphatidylserine and phosphatidic acid) are only detected in negative mode analyses when complex samples such as plasma are studied. Therefore, not having that information in this manuscript causes a hole that needs to be filled. One other small comment would be that the PA trend line is not apparent in Figure 2. Other than these critiques, the manuscript was very well written and would definitely strengthen the lipidomic analyses that can be performed to date. If both ionization modes are presented in the revised manuscript, I feel this would be a fundamental paper in Nature Communications for people performing lipid analyses and identifications.

Reviewer #2 (Remarks to the Author):

The manuscript by McLean et al. presents the largest compilation of lipid collision cross section values, as determined by ion mobility-mass spectrometry, to date. These collision cross sections are useful in the complex task of metabolite (lipid) structural identification, this being recognized widely as the main bottleneck in current metabolomic/lipidomic efforts. Typically, metabolomic/lipidomic experiments employ high resolution liquid chromatography-mass spectrometry (LC-MS) as the method for interrogating the component of the investigated samples, followed by a complex workflow for mining the data using peak alignment, peak picking, integration and de-replication (de-adduction, de-isotoping) techniques. These steps can be largely automated in state-of-the art platforms. However, the next step typically consists in assigning chemical structures to a small subset (e.g. a potential biomarker panel) or a large subset of species (e.g. those molecules selected through a volcano plot). This stage can typically consume months of time for even the most skilled analyst, and requires extensive training in the basic tools typically used for metabolite identification, such as mass spectral interpretation, understanding of gas-phase fragmentation mechanisms,

metabolite database searches, etc. This task can quickly balloon and leave many metabolites unidentified, preventing biological interpretation of metabolomics (lipidomic) results through pathway enrichment analysis. Lipids, in particular, follow combinatorial rules that lead to a large number of species being isobaric (indistinguishable by their elemental formula). Even with tandem MS experiments, many times results are unsatisfactory due to the fact that isobaric lipids are co-selected leading to mass spectra that are a composite of the various precursors. Even though nuclear magnetic resonance (NMR) spectroscopy is one of the mainstays in terms of small molecule identification, it typically does not achieve the levels of sensitivity and speed to be compatible with online LC-MS lipidomics experiments. The approach presented by McLean and coworkers tackles the specific problem of better lipid identification in lipidomic experiments by using collision cross sections (CCS) values as an additional molecular descriptor that enables distinction of lipid species based on their gas phase conformation. As such, this work is viewed as highly significant. It is also quite innovative: despite the fact that other authors have presented CCS databases for lipids (perhaps the best example is that of Astarita et al., Anal. Chem. 87, 2, 1137-1144), the database presented here is by far the largest and most complete, providing information that will be incredibly valuable to anybody involved in the rapidly evolving field of metabolomics/lipidomics, the newest of the omics. The manuscript, overall, is well written and very clear, and the data presented in an easy to understand way that will likely be attractive to a wide readership. Overall, I am in favor of publication conditional to addressing the specific points brought up below:

Motivation and Background:

-This section is comprehensive enough, but could be polished a little in terms of style. Sentences do not flow well. Despite this, the motivation provided is sufficient to make a strong case for the presented results. Perhaps, suggest adding a few other references (the work of Yu Xia is of particular relevance here, see doi.org/10.1073/pnas.1523356113, for example).

-Work on prediction of CCS values by various means is growing in the literature, see for example Chem. Commun., 2017,53, 7624-7627. This should be mentioned as an alternative approach to identifying lipids.

Results and discussion

-Figure 2 clearly supports the statement that CCS values enabled distinction of lipids into two main classes, namely sphingolipids and glycerophospholipids. One could argue that it is also possible to distinguish, in most cases, these two families by m/z alone, at high enough resolving power. The authors should bring this point forward and perhaps present a case where m/z alone would not allow this distinction, so as to showcase the innovative aspects of this work and the advantages of collecting CCS information.

-I find Figure S1 intriguing but difficult to understand/interpret. Can the authors clarify? Perhaps elaborate in the caption?

-I think it could be useful to provide the information on the various regression lines shown throughout the manuscript in some sort of shape or form. Perhaps a spreadsheet?

-The example provided for the unknown lipid in Fig. 4(C) (802.62 212 Da, 290.3 Å2) is a good one, it illustrates well the use of CCS for identification of metabolites. However, the scenario provided is not too realistic. Most lipidomic experiments also rely on MS/MS information for identification (m/z alone is not sufficient, see doi: 10.1186/1471-2105-8-105). Can the authors provide additional data/experiments with MS/MS information for the species in question? The expected MS/MS fragmentation spectrum for the discussed species should be very different and should be able to tell them apart.

-Lipid mixture analysis: The authors state that "This in turn allows accurate mass and DTCCSN2 information to be obtained. Using the DTCCSN2 information, more confident identifications of the components in this narrow mass window can be made". This is an accurate statement, but it may be worth showing exactly how the assignment is done (perhaps in supplementary information?). How do the trendlines inform the decision? Is there any ambiguity? Etc etc.

Methods:

-"All 2D IM-MS spectra was" should be "All 2D IM-MS spectra were".

-"source" should be "ion source"

-Please comment if any of the ion optics settings had a major influence on CCS measurements do to ion heating. This could be useful for readers not familiar with the Agilent instruments, and coming from another platforms, such as traveling wave IM-MS.

-Tetraalkylammonium salts are notorious for inducing ion suppression. Please indicate if this was observed at the concentration levels tested, or not.

-In the supplemental table, clarify that the molecular formulae are for the adduct ions in the column header.

-In the supplemental table provide measured and theoretical m/z with 4 decimal places, as customary in high resolution MS. Also in this table, please indicate the meaning of each column header so the table is self-explanatory.

-Because continuous infusion was used for performing the experiments, a large fraction of the lipids studied is ionized as the [M+Na]+ adduct, which is to be expected. A large fraction of lipidomic experiments is performed by LC-MS, so the prevalence of sodium adducts is typically much lower (almost none, depending on sample complexity and sodium content). CCS values for protonated species will likely be more valuable than sodiated species in those scenarios. Please comment on the difference expected in gas-phase packing (and therefore in CCS values) in this scenario.

-No MS/MS was used for identity validation purposes. Why not? This would strengthen the robustness of the database entries.

**Ion Mobility Conformational Lipid Atlas for High Confidence Lipidomics**

resubmission to *Nature Communications*

**Responses to the Comments from Referee 1**

***Response:*** *We greatly appreciate the time this Referee has dedicated in providing critical review and constructive recommendations for our manuscript. Please find our specific responses noted below.*

**Referee 1 Comment 1:** In this manuscript, the authors compiled a structural database of 354 mass-resolved collision cross section (CCS) values within sphingolipid and glycerophospholipid categories using IM-MS in positive ionization mode. The authors then used this information to analyze the primary differences between lipid categories. In their assays, acyl tail length and degree of unsaturation were found to be the primary structural descriptors for the different lipid categories and classes. The data included in this manuscript is novel as no group to date has done such an extensive characterization of lipid structures with IM-MS. However, I feel that the data from negative ionization mode also needs to be included for the manuscript to fully influence lipidomic analyses. Many of the lipids studied (i.e. phosphatidylserine and phosphatidic acid) are only detected in negative mode analyses when complex samples such as plasma are studied. Therefore, not having that information in this manuscript causes a hole that needs to be filled.

***Response:*** *We agree that negative ion data is important, and as such we have included new measurements for negative ions observed from all five lipid samples that we initially investigated (PE, PC, PS, SM, GlcCer). We note that no PA or Cer features were observed in negative mode. The negative mode data has been incorporated throughout the manuscript and in the updated figures included in our resubmission.*

**Referee 1 Comment 2:** One other small comment would be that the PA trend line is not apparent in Figure 2.

***Response:*** *We thank the Referee for noting this lack of clarity and have amended Figure 2 for better visibility of the PA trend line.*

**Referee 1 Comment 3:** Other than these critiques, the manuscript was very well written and would definitely strengthen the lipidomic analyses that can be performed to date. If both ionization modes are presented in the revised manuscript, I feel this would be a fundamental paper in Nature Communications for people performing lipid analyses and identifications.

***Response:*** *We appreciate these supportive comments from the Referee.*

**Responses to the Comments from Referee 2**

***Response:*** *We greatly appreciate the time this Referee has dedicated in providing critical review and constructive recommendations for our manuscript. Please find our specific responses noted below.*

**Referee 2 Comment 1:** The manuscript by McLean et al. presents the largest compilation of lipid collision cross section values, as determined by ion mobility-mass spectrometry, to date. These collision cross sections are useful in the complex task of metabolite (lipid) structural identification, this being recognized widely as the main bottleneck in current metabolomic/lipidomic efforts. Typically, metabolomic/lipidomic experiments employ high resolution liquid chromatography-mass spectrometry (LC-MS) as the method for interrogating the component of the investigated samples, followed by a complex workflow for mining the data using peak alignment, peak picking, integration and de-replication (de-adduction, de-isotoping) techniques. These steps can be largely automated in state-of-the art platforms. However, the next step typically consists in assigning chemical structures to a small subset (e.g. a potential biomarker panel) or a large subset of species (e.g. those molecules selected through a volcano plot). This stage can typically consume months of time for even the most skilled analyst, and requires extensive training in the basic tools typically used for metabolite identification, such as mass spectral interpretation, understanding of gas-phase fragmentation mechanisms, metabolite database searches, etc. This task can quickly balloon and leave many metabolites unidentified, preventing biological interpretation of metabolomics (lipidomic) results through pathway enrichment analysis. Lipids, in particular, follow combinatorial rules that lead to a large number of species being isobaric (indistinguishable by their elemental formula). Even with tandem MS experiments, many times results are unsatisfactory due to the fact that isobaric lipids are co-selected leading to mass spectra that are a composite of the various precursors. Even though nuclear magnetic resonance (NMR) spectroscopy is one of the mainstays in terms of small molecule identification, it typically does not achieve the levels of sensitivity and speed to be compatible with online LC-MS lipidomics experiments. The approach presented by McLean and coworkers tackles the specific problem of better lipid identification in lipidomic experiments by using collision cross sections (CCS) values as an additional molecular descriptor that enables distinction of lipid species based on their gas phase conformation. As such, this work is viewed as highly significant. It is also quite innovative: despite the fact that other authors have presented CCS databases for lipids (perhaps the best example is that of Astarita et al., Anal. Chem. 87, 2, 1137-1144), the database presented here is by far the largest and most complete, providing information that will be incredibly valuable to anybody involved in the rapidly evolving field of metabolomics/lipidomics, the newest of the omics. The manuscript, overall, is well written and very clear, and the data presented in an easy to understand way that will likely be attractive to a wide readership. Overall, I am in favor of publication conditional to addressing the specific points brought up below:

Motivation and Background:

-This section is comprehensive enough, but could be polished a little in terms of style. Sentences do not flow well. Despite this, the motivation provided is sufficient to make a strong case for the presented results. Perhaps, suggest adding a few other references (the work of Yu Xia is of particular relevance here, see doi.org/10.1073/pnas.1523356113, for example).

-Work on prediction of CCS values by various means is growing in the literature, see for example Chem. Commun., 2017,53, 7624-7627. This should be mentioned as an alternative approach to identifying lipids.

***Response:*** *We greatly appreciate the Referee's perspectives here and agree with their suggestions. To address the technical shortcomings of our manuscript, we have significantly reworked the structure and language of the Motivation and Background section, as noted in the "tracked changes" document included in our resubmission. We have also added the two references suggested by the Referee, as well as two additional works in the area of CCS prediction and annotation: Hancock et al. 2017 (DOI: 10.1016/j.ab.2016.09.014), and Picache et al. 2018 (DOI: 10.1039/c8sc04396e.).*

**Referee 2 Comment 2:** Results and discussion

-Figure 2 clearly supports the statement that CCS values enabled distinction of lipids into two main classes, namely sphingolipids and glycerophospholipids. One could argue that it is also possible to distinguish, in most cases, these two families by m/z alone, at high enough resolving power. The authors should bring this point forward and perhaps present a case where m/z alone would not allow this distinction, so as to showcase the innovative aspects of this work and the advantages of collecting CCS information.

***Response:*** *We agree with the Referee that, with sufficiently high resolving power, lipid classes can be distinguished by mass measurement alone. As such, we have strengthened the language describing Figure 2 and present a new case in our revised text in which m/z alone would not be sufficient to differentiate lipid class.*

**Referee 2 Comment 3:** -I find Figure S1 intriguing but difficult to understand/interpret. Can the authors clarify? Perhaps elaborate in the caption?

***Response:*** *We agree with the Referee here that Figure S1 is unclear, and thus (1) we have revised the text in the manuscript (under the Cation Forms section) to better describe Fig S1, (2) renamed the section to "Ion Forms" in order to better accommodate the negative mode data, and (3) we have expanded upon the caption for Fig S1 (as suggested by the Referee) to clarify the figure and its significance.*

**Referee 2 Comment 4:** -I think it could be useful to provide the information on the various regression lines shown throughout the manuscript in some sort of shape or form. Perhaps a spreadsheet?

***Response:*** *Thank you. We agree that additional transparency in the linear regression data analysis would be beneficial to readers of the manuscript. To address this suggestion, we have added a new table (Table S3) in the supplemental information which presents the data for the regression lines in Figure 2. Although we originally included summary data for the regression lines from Figure 4 and S2, we have also now included a new table in the supplemental information (Table S4) with the individual data for each regression analysis.*

**Referee 2 Comment 5:** -The example provided for the unknown lipid in Fig. 4(C) (802.62 212 Da, 290.3 Å2) is a good one, it illustrates well the use of CCS for identification of metabolites. However, the scenario provided is not too realistic. Most lipidomic experiments also rely on MS/MS information for identification (m/z alone is not sufficient, see doi: 10.1186/1471-2105-8-105). Can the authors provide additional data/experiments with MS/MS information for the species in question? The expected MS/MS

fragmentation spectrum for the discussed species should be very different and should be able to tell them apart.

***Response:*** *We agree with the Referee that verification of the assigned lipid identity via an orthogonal analytical technique, such as tandem MS/MS, would strengthen the argument. As the Referee suggested, we attempted to fragment the lipid at m/z 802.62 (identified as GlcCer 40:03 + Na), however, this particular spectral feature is both low abundance and partially overlaps in mass and mobility with another feature, making it difficult to isolate in either dimension, and thus the quality of the MS/MS spectra was poor. Subsequently, we were unable to identify any structurally-informative fragment ions in the ion fragmentation data. We note that metal-adducted lipid species are challenging to fragment, as the lowest energy pathway is the ejection of the metal, resulting in a neutral lipid fragment.*

**Referee 2 Comment 6:** -Lipid mixture analysis: The authors state that "This in turn allows accurate mass and DTCCSN2 information to be obtained. Using the DTCCSN2 information, more confident identifications of the components in this narrow mass window can be made". This is an accurate statement, but it may be worth showing exactly how the assignment is done (perhaps in supplementary information?). How do the trendlines inform the decision? Is there any ambiguity? Etc etc.

***Response:*** *We agree with the Referee's suggestion here that more information regarding the assignment of identifications using the trend lines would strengthen the manuscript. To address this point, we have included an example of how trendline information can help to identify ambiguous features in Fig. 4(E) with accompanying text in the section "Identification by CCS." To clarify for future readers, we have also added the following statement to the "Lipid Mixture Analysis" section: "Identifications for the mixture were made by matching to known lipid features in the individually analyzed lipid extracts."*

**Referee 2 Comment 7:** Methods:

-"All 2D IM-MS spectra was" should be "All 2D IM-MS spectra were".

***Response:*** *Thank you. This correction has been made.*

**Referee 2 Comment 8:** -"source" should be "ion source"

*This designation has been incorporated throughout.*

**Referee 2 Comment 9:** -Please comment if any of the ion optics settings had a major influence on CCS measurements do to ion heating. This could be useful for readers not familiar with the Agilent instruments, and coming from another platforms, such as traveling wave IM-MS.

***Response:*** *The Referee raises an important point here as many IM-MS users are more familiar with the traveling wave platform, where ion heating can influence the CCS measurement. To address this point, we have included a statement in our manuscript under the section entitled "Prediction of CCS" which states that ion optical effects on CCS were observed in our prior work, but were not related to ion heating.*

**Referee 2 Comment 10:** -Tetraalkylammonium salts are notorious for inducing ion suppression. Please indicate if this was observed at the concentration levels tested, or not.

***Response:*** *We thank the Referee for this insight. In our original data, we observed strong lipid signal despite the inclusion of TAA salts. We have added a sentence in the "Calibration Methods" section which*

*states that we conducted experiments without the TAA salts and obtained similar results which suggests that no significant ion suppression is occurring at the concentrations utilized in our work.*

**Referee 2 Comment 11:** -In the supplemental table, clarify that the molecular formulae are for the adduct ions in the column header.

***Response:*** *We thank the Referee for raising this point. We agree that the use of adduct ion formulas is unconventional and unclear in our original submission, so we have chosen to replace all of the ionic formulas listed in Table S5 with neutral molecular formulas, and have labeled the column appropriately as "Neutral Formula".*

**Referee 2 Comment 12:** -In the supplemental table provide measured and theoretical m/z with 4 decimal places, as customary in high resolution MS. Also in this table, please indicate the meaning of each column header so the table is self-explanatory.

***Response:*** *We thank the Referee for this suggestion. We have provided the m/z measurements to 4 decimal places. We have also added a description of the column headers in the table captions for all supplementary tables.*

**Referee 2 Comment 13:** -Because continuous infusion was used for performing the experiments, a large fraction of the lipids studied is ionized as the [M+Na]+ adduct, which is to be expected. A large fraction of lipidomic experiments is performed by LC-MS, so the prevalence of sodium adducts is typically much lower (almost none, depending on sample complexity and sodium content). CCS values for protonated species will likely be more valuable than sodiated species in those scenarios. Please comment on the difference expected in gas-phase packing (and therefore in CCS values) in this scenario.

***Response:*** *We are grateful for the Referee's perspective regarding the different adduct types observed and their potential effects on the measured CCS. While we have also analyzed these lipid extracts with LC-MS, this data is not included in this study, but we do note here that a significant number of sodiated ions were also observed for LC-MS, specifically, we observed approximately three $[M+Na]^+$ features for every four $[M+H]^+$ adducts. To address the Referee's comment, we have included Supplementary Fig. 1 which summarizes the effect of various adducts on lipid gas-phase packing , projected as a measure of the change in CCS relative to the lipid ion form. This relationship is also addressed in the section "Ion Forms" (previously "Cation Forms"). The second paragraph includes the quantitative relationship: "In general for positively charged ions, $[M+Na]^+$, $[M+K]^+$, and $[M+2Na-H]^+$ features increased ${}^{DT}CCS_{N2}$ over $[M+H]^+$ by 2.5 ± 2.0 Å$^2$, 4.7 ± 1.2 Å$^2$, and 5.6 ± 1.4 Å$^2$ in 43, 15, and 26 cases, respectively." We have also expanded upon the text regarding Fig S1 and its caption to clarify these discussion points and their significance.*

**Referee 2 Comment 14:** -No MS/MS was used for identity validation purposes. Why not? This would strengthen the robustness of the database entries.

***Response:*** *We thank the Referee for this suggestion. To address this comment in part, we have added select ion fragmentation data as Supplemental Fig. 5 (and a reference to it in the text at the end of the "Quantitative Mobility-Mass Correlations to Construct a Lipid Atlas" section). We note here and in the manuscript that we did acquire data-independent ion fragmentation spectra for all lipid extracts, which yielded some information regarding lipid class, however, the quality of the DIA spectra is rather poor and thus provided only limited information to support lipid identifications. Targeted MS/MS experiments*

*would yield higher-quality fragmentation information, however, we did not pursue these studies as we were confident in our assignments based on the mass measurement and CCS data.*

REVIEWERS' COMMENTS:

Reviewer #1 (Remarks to the Author):

The authors have addressed all of my original concerns in this new version of the manuscript. I feel the revised manuscript will provide the structural lipid characteristics needed by the scientific community for more confident lipid identifications in both positive and negative polarity analyses. I feel this manuscript is now be accepted for publication in Nature Communications.

Reviewer #2 (Remarks to the Author):

The authors have favorably and very positively responded to the initial set of critiques, and the current version of the manuscript has greatly improved. I recommend this article is now published as is.
